# Statistics of Symphysiotomy

**Published:** 1883-04

**Authors:** 


### STATISTICS OF SYMPHYSIOTOMY.

Dr. Robert P. Harris publishes in the *American Journal of the Medical Sciences* for January, 1883, a careful analysis of the statistics of symphysiotomy, with comparative tables of the early and later cases, showing that the operation has been more frequently performed in Italy in the last seventeen years than in the previous eighty. In the first table, extending up to 1858, out of 70 cases there was a maternal mortality of 70 per cent., and a fœtal mortality of 67 per cent. The second table begins with the resuscitation of this operation in Naples, in 1866, and as far as he has been able to learn, there have been 53 operations in that city, saving 43 women and 42 children. From the report of Prof. Morisani, by whom most of these operations were performed, we learn that—

1. All of the fifty operations (in table 2) were performed upon rachitic subjects, whose pelves were generally flattened antero-posteriorly. In four or five instances the pelves were simply dwarfed in dimensions. There was no case of rostrate pelvis, as *Malacosteon* is very rarely met with in Naples.

2. Version was not resorted to except in the transverse positions. The forceps were applied in about one-fourth of the cases.

3. The separation of the pubes amounted to about 2 inches (50 mm.), which was obtained without any effort, and without producing any lesion of the sacro-iliac synchondroses.

4. The immovable dressing secured the firm union of the symphysis pubis in all the cases that recovered.

5. The women had good health after the operation.

6. There were no malformed infants. Nearly all of the children were sent to the Foundling Hospital to be taken care of.

7. *Phlegmasia alba dolens* did not occur in any of the women.

8. There were no pelvic lesions left, as a sequal of the operation, with the exception of one case of iliac phlegmon.

9. Vesico-vaginae fistula occurred in but one case, and this was easily cured by an operation.

